# Supplementary material for: Absolute Membrane Potential Recording with ASAP-Type Genetically Encoded Voltage Indicators Using Fluorescence Lifetime Imaging
Source: ACS Chem Neurosci. 2025 Nov 22;16(24):4636–46. doi: 10.1021/acschemneuro.5c00670 (PMC12715753; doi:10.1021/acschemneuro.5c00670)
Supplement: Supplementary file 1 [file cn5c00670_si_001.pdf]

Absolute Membrane Potential Recording with ASAP-Type Genetically Encoded Voltage Indicators Using Fluorescence Lifetime Imaging

Anagha Gopalakrishnan Nair[a], Marko Rodewald[b†], Hyeonsoo Bae[b], Philipp Rühl[a], Jürgen Popp[b,c], Michael Schmitt[c], Tobias Meyer-Zedler[b], Stefan H. Heinemann[a,*]

Supporting Information

[a] *Center for Molecular Biomedicine, Department of Biophysics, Friedrich Schiller University Jena and Jena University Hospital, Hans-Knöll-Straße 2, 07745 Jena, Germany*

[b] *Leibniz Institute of Photonic Technology, Member of Leibniz Health Technologies, Member of the Leibniz Centre for Photonics in Infection Research (LPI), Albert-Einstein-Straße 9, 07745 Jena, Germany*

[c] *Institute of Physical Chemistry (IPC) and Abbe Center of Photonics (ACP), Friedrich Schiller University Jena, Member of the Leibniz Centre for Photonics in Infection Research (LPI), Helmholtzweg 4, 07743 Jena, Germany, the Cluster of Excellence Balance of the Microverse, Friedrich Schiller University, Jena*

† *Current address: Department of Physics, Polytecnico di Milano, Piazza Leonardo da Vinci 32, 20133 Milan, Italy*

* Corresponding author
Center for Molecular Biomedicine, Department of Biophysics, Friedrich Schiller University Jena and Jena University Hospital, Hans-Knöll-Straße 2, 07745 Jena, Germany
stefan.h.heinemann@uni-jena.de
ORCID: 0000-0002-4144-0251

SUPPORTING METHODS

Determination of the molecular brightness. To estimate the molecular brightness of the GEVI constructs (Figure 2C), they were expressed in HEK293T cells as fusion proteins with mKate2, thus placing mKate2 at the intracellular face of the membrane, while the cpGFP part of the GEVI was located on the extracellular side (Figure S2A). The voltage of the transiently transfected cells was clamped to -120 mV, and green (GEVI) and red (mKate2) fluorescence were measured photometrically using a Polychrome V monochromator (TILL Photonics, Grafelfing, Germany) and a ProgRes MF cool camera (JenOptik, Jena, Germany). For cpGFP imaging, the excitation wavelength was 480 nm with the following filter set: excitation 492/SP, beamsplitter FT495, emission BP525/50. For mKate2 imaging, the excitation wavelength was 530 nm with the following filter set: excitation BP510-560, beamsplitter FT580, emission LP590. The microscope was an Axio Observer with a 40x EC Plan Neofluar oil-immersion objective (1.3 NA, Zeiss). The relative molecular brightness was defined as the F_{green} / F_{red} ratio at -120 mV.

Fluorescence Lifetime Image Processing. To generate fluorescence lifetime images, the second half of the acquired frames were binned using an all-photon filter and individual pixels were fitted with a two-component exponential decay model with deconvolution of the instrument response function (IRF). This analysis yielded two lifetime images (Tau1, Tau2 or τ_{fast}, τ_{slow}) and their corresponding intensity images (Int1, Int2). All images were exported as ImageJ-TIFF files with fixed scaling: intensity images were scaled by a factor of 1.0 and lifetime images by 0.001.

An intensity-based thresholding approach was applied to isolate pixels corresponding to the cell membrane while excluding background and noisy regions, without requiring manually drawn ROIs. The threshold was automatically determined by selecting the intensity value that included the top 1000 brightest pixels. A total intensity image (IntSum) was computed by summing Int1 and Int2, representing the total photon count per pixel. A binary mask was generated by applying a user-defined threshold to IntSum: pixels above the threshold were included and those below were excluded. For each valid pixel, an intensity-weighted average lifetime was calculated. Although we consistently used intensity-weighted lifetime estimates (also termed fraction-weighted), we also show in Figure S2 for rEstus-NI the distribution of the intensity-weighted lifetimes along with the decomposition into the fast and slow components: τ_{fast} and τ_{slow}.

For the analysis of experiments involving many cells, the photon arrival times from these cells (from the second half of the frames, i.e., the last 200 frames out of a total of 400 frames) were binned. A manual ROI was drawn along the cell membrane, and the data were then fitted using a two-component deconvolution fit model. For examples, see Figure S1 and S3.

We selected a two-component decay model as compared to a single-exponential decay based on the consistently better fit results, as shown in Figures S1 and S3. Furthermore, in Figure S4 it is demonstrated that the FLIM recording noise can be diminished by constraining the fast and slow lifetime constants (τ_{fast} and τ_{slow}) to their means from a series of image frames, and only varying their amplitudes.

Analysis of Time Dependence. Kinetics in F in response to step-like voltage excursions from -60 mV were described using double-exponential functions for the “on” (after t_0) and “off” processes (after t_1).

The slow time constants for "on" ($\tau_{s,on}$) and "off" ($\tau_{s,off}$) were freely fitted, while the resolution-limited fast time constant (τ_f) and the relative fraction of the slow component (r_s) were identical for both the "on" and "off" directions:

$$\frac{F(t < t_0)}{F_{-60mV}} = F_{-60mV} \qquad \text{Eq. S2}$$

$$\frac{F(t_0 \le t < t_1)}{F_{-60mV}} = F_{-60mV} + (F_{\max} - F_{-60mV})\left\{\left(1 - r_s\right)_1 \left(1 - \mathrm{e}^{-(t-t_0)/\tau_f}\right) + r_s\left(1 - \mathrm{e}^{-(t-t_0)/\tau_{s,on}}\right)\right\}$$

$$\frac{F(t \ge t_1)}{F_{-60mV}} = F_{t_1} - (F_{t_1} - F_{-60mV})\left\{\left(1 - r_{off}\right)\left(1 - \mathrm{e}^{-(t-t_0)/\tau_f}\right) + \left(1 - r_{off}\right)\left(1 - \mathrm{e}^{-(t-t_0)/\tau_{s,off}}\right)\right\}$$

with $r_{off} = r_s \dfrac{F_{\max} - F_{-60mV}}{F_{t_1} - F_{-60mV}}$.

SUPPORTING FIGURES AND TABLES

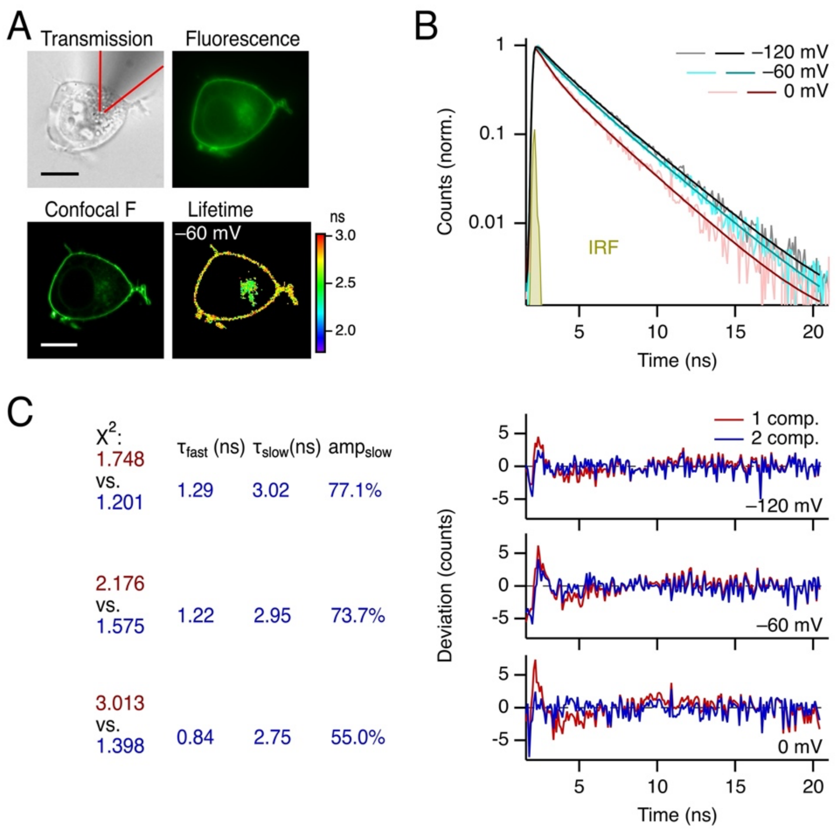

Supporting Figure 1. FLIM of HEK293T cells expressing rEstus. (A) Images of a HEK293T cell expressing rEstus with a patch-clamp pipette attached: (i) transmission image with the red lines indicating the patch-clamp pipette, which is out of focus; (ii) raw fluorescence with excitation at 488 nm; (iii) confocal fluorescence intensity of the membrane; (iv) mean intensity-weighted fluorescence lifetime of 20 consecutive images similar to (iii) after masking pixels with too small intensity. Scale bar: 10 µm. Note that the intracellular components have a fluorescence lifetime unrelated to the plasma membrane potential and, hence, have to be eliminated. (B) Photon counts (means of 20 consecutive frames, normalized to the maximum) originating from a membrane-delimiting ROI, as a function of time after triggering the excitation in response to the indicated instrument response function (IRF) for the specified voltages. Thin traces are raw data, and thick lines are double-exponential fits after deconvolution of the IRF. (C) Analysis of the data fits from (B): Deviation between a single-component fit (red) and a double-component fit (black) from the data for the indicated voltages. The X^2 deviation is given along with the fitted lifetime constants and the relative amplitude of the slow component.

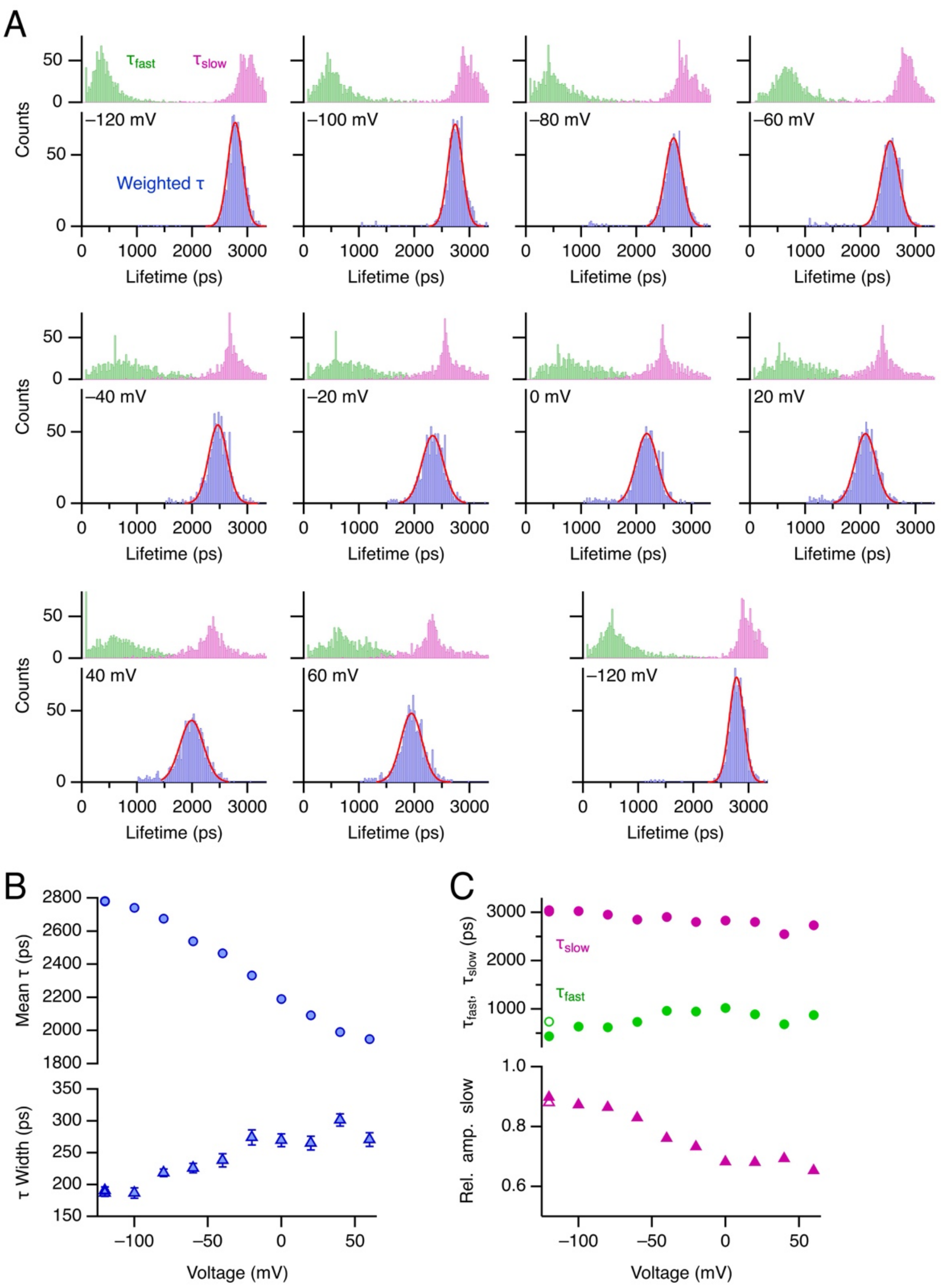

Supporting Figure 2. Distribution of fluorescence lifetime values for rEstus-NI. (A) Histograms of fluorescence lifetimes for the indicated voltages from the images shown in Figure 1G. Bin width = 25 ps. (*top*) Histograms of the fast (green) and the slow (magenta) lifetime components; (*bottom*) histograms of the intensity-weighted lifetime with superimposed Gaussian fits (red). (B) Gaussian fit results from (A): mean τ_{fl} of the distribution (*top*) and half-width (*bottom*) as a function of voltage; error bars are ±95% confidence interval. (C) Individual mean fast and slow time constants (*top*) and the relative amplitude of the slow component (*bottom*) as a function of voltage. Open symbols are from the lifetime determination at -120 mV at the end of the calibration.

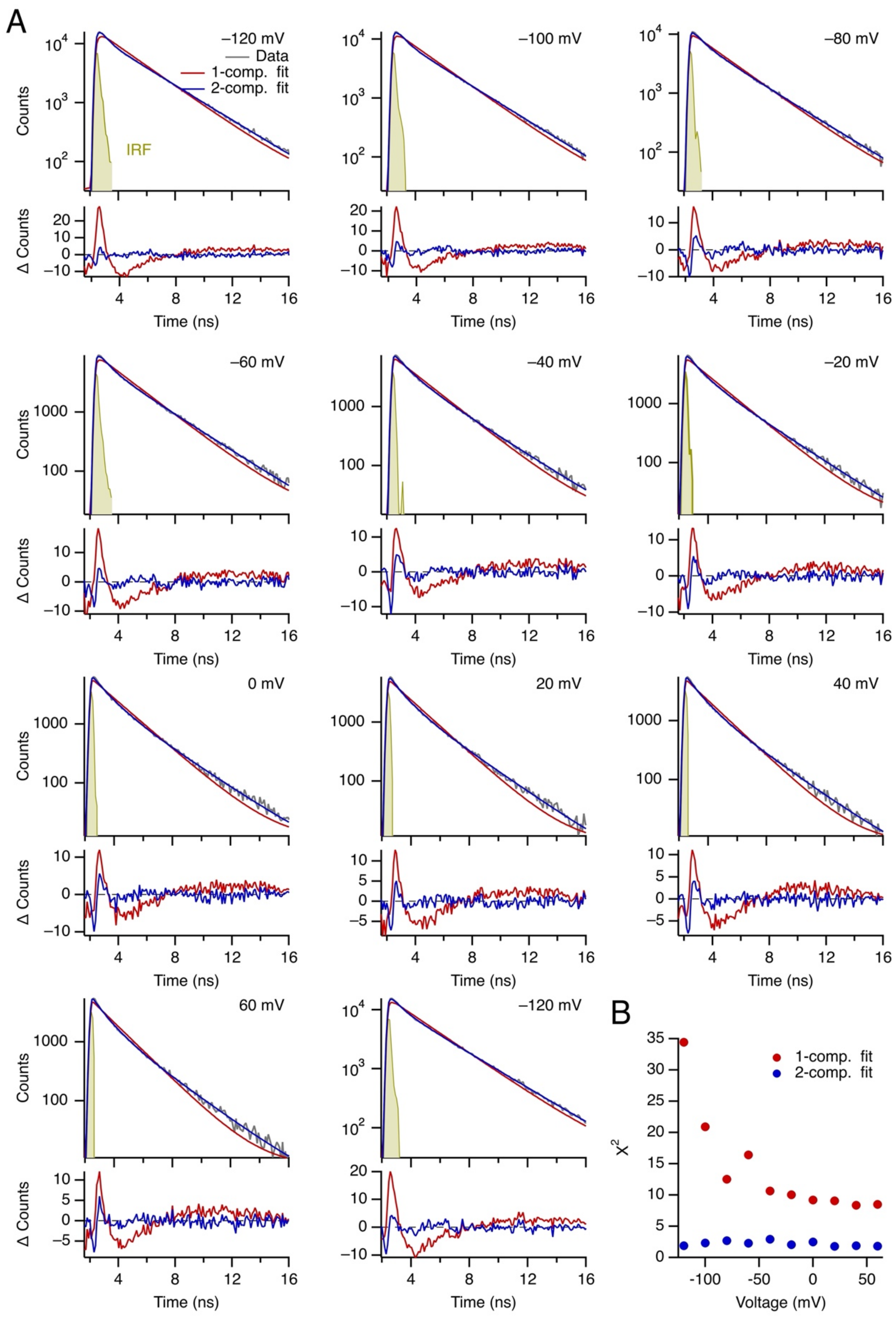

Supporting Figure 3. Fluorescence decay fits for rEstus-NI. (A) *top,* Fluorescence decay of rEstus-NI in voltage-clamped HEK293T cells from membrane-delimiting ROIs of data shown in Figure 1G, accumulated over 20 consecutive frames. The voltage is indicated. Data, shown in gray, were fit with single- (red) and double-exponential functions (blue) considering the convolution with the laser excitation timecourse (IRF, instrument response function). *bottom*, Residuals between data and fit for single- and double-exponential fits. (B) X^2 deviation of the fit in (A) as a function of voltage.

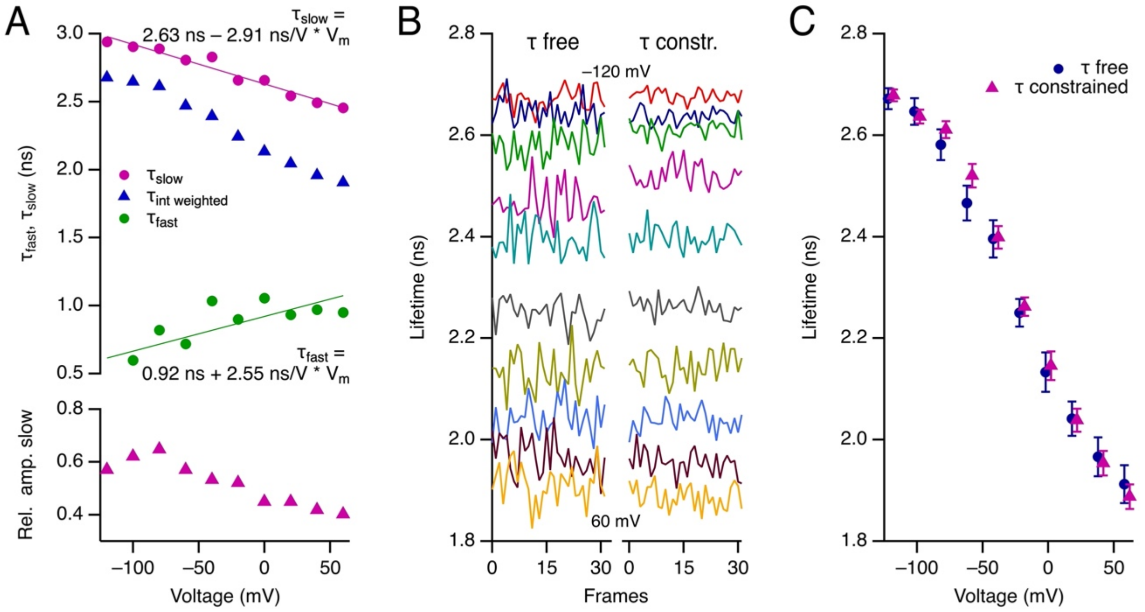

Supporting Figure 4. Analysis of the two-component fits of rEstus-NI data shown in Figure S3. (A) *top*, Intensity-weighted lifetime (blue), as well as the time constants of the fast (green) and slow (magenta) component. The superimposed lines are linear fits to the fast and slow lifetime constants with the indicated results. *bottom*, Relative slow amplitude of the two-component decay fit. (B) Comparison of time-lapse determinations of intensity-weighted lifetimes based on individual image frames for which the fluorescence decays were fitted either freely (*left*) or by constraining the fast and slow lifetime constants to the values obtained from the fits in (A) (*right*). Data are from one rEstus-NI-expressing cell, clamped to -120 mV (*top*) to 60 mV (*bottom*) in steps of 20 mV. (C) Analysis of the lifetime traces shown in (B) as a function of voltage: symbols are mean values ± SD. Data points are offset ± 2 mV to enhance visibility. The standard deviation averaged for all voltages examined were 31.3 ps for the free fit and 18.7 ps for the constrained fit.

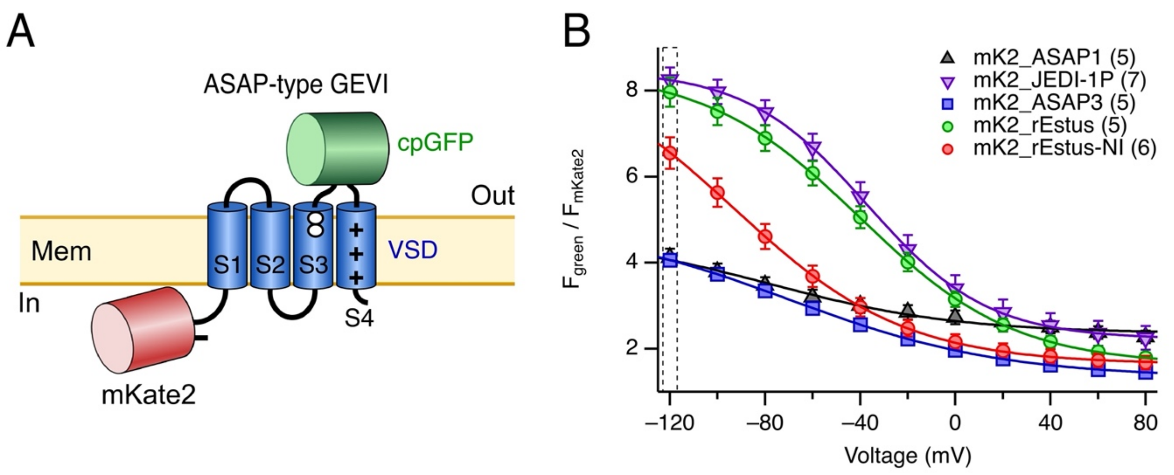

Supporting Figure 5. mKate2-GEVI fusion for brightness determination. (A) Scheme of the mKate2-GEVI fusion constructs used for brightness calibration. The GEVIs of the ASAP family have in common a voltage-sensing domain (VSD) consisting of four transmembrane helices (S1-S4, blue) and a circularly permuted GFP (cpGFP) inserted on the extracellular side between S3 and S4. The white circles in S3 mark the approximate positions of residues G138 and T141 of rEstus. (B) Voltage dependence of the fluorescence ratio F_{green} / F_{mKate2}, originating from the cpGFP moiety of the voltage-dependent GEVIs and the N-terminally fused mKate2, respectively. The mKate2 fluorescence serves as a calibration signal to compare the brightness of the indicated GEVIs at -120 mV (see Figure 2C). Data are means ± SEM, with *n* in parentheses. Superimposed curves are fit results according to Eq. 1; the resulting parameters are listed in Table S1.

Supporting Table 1. Voltage dependence of GEVI fluorescence relative to mKate2 fluorescence. Results of the data fits as shown in Figure S5 according to Eq. 1. Note that the fit for ASAP1 was constrained regarding to a reasonable steepness (k_h = 40 mV) because, even at -120 mV, the fluorescence is far from being saturated. Errors are 95% confidence intervals.

mKate2 fusion with	Max. F_{green} / F_{red}	Offset (%)	V_{hF} (mV)	k_{hF} (mV)	n
ASAP1	4.66 ± 0.19	50.6 ± 1.9	-78.3 ± 8.9	40, constr.	5
JEDI-1P	8.48 ± 0.29	26.3 ± 0.3	-37.2 ± 0.4	25.4 ± 0.4	7
ASAP3	4.96 ± 0.02	26.8 ± 0.2	-69.9 ± 0.6	45.0 ± 0.4	5
rEstus	8.49 ± 0.04	19.1 ± 0.3	-40.3 ± 0.4	32.6 ± 0.5	5
rEstus-NI	8.87 ± 0.16	18.5 ± 0.1	-92.9 ± 1.7	35.8 ± 0.7	6

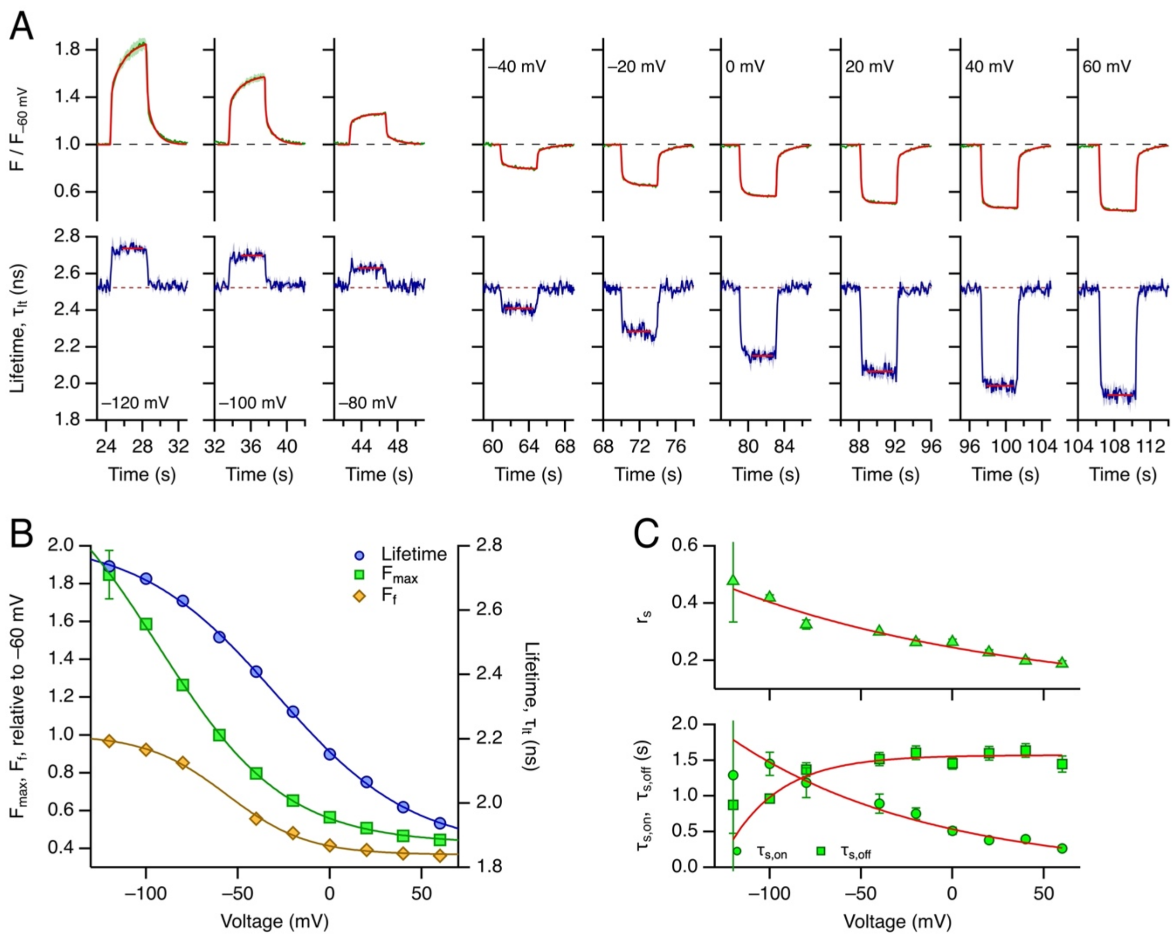

Supporting Figure 6. Kinetics of voltage-step responses in fluorescence and fluorescence lifetime of rEstus-NI. (A) Responses of *F* normalized to *F* at -60 mV (*top*) and fluorescence lifetime (τ_{lt}) (*bottom*) to voltage steps to the indicated voltage from and to -60 mV (data from Figure 1E). The *F* data are superimposed with fit functions according to Eq. S2, thus comprising double-exponential "on" and "off" phases with a common fast time constant (τ_f, 70 ms) and a common fraction of the fast component (r_s). The red lines superimposed on the lifetime data show the data range used for determining the mean. (B) Voltage dependence of the maximal fluorescence intensity (F_{max}), the intensity of the fast component only (F_f), and the fluorescence lifetime with superimposed fits according to Eq. 1. For the resulting parameters, see Table S2. (C) Results of the kinetics analysis showing the voltage dependence of the fraction of the slow component (r_s, *top*), and the time constants of the slow component for the voltage step from ($\tau_{s,on}$) and the return to -60 mV ($\tau_{s,off}$). The superimposed curves are fitted single-exponential functions to indicate the trend of voltage dependence.

Supporting Table 2. Voltage dependence of rEstus-NI fluorescence components based on double-exponential fits according to Eq. S2. The table contains the parameters describing the data fits according to Eq. 1 shown in Figure S6B.

Parameter	Δa	a_{inf}	V_h (mV)	k_h (mV)
Lifetime, τ_{lt}	0.96 ± 0.03 ns	1.86 ± 0.02 ns	-29.2 ± 1.4	37.3 ± 1.9
F_{max}	2.09 ± 0.09	0.43 ± 0.01	-93.4 ± 2.9	34.8 ± 1.4
F_f	0.63 ± 0.02	0.37 ± 0.01	-56.0 ± 2.3	21.9 ± 1.7

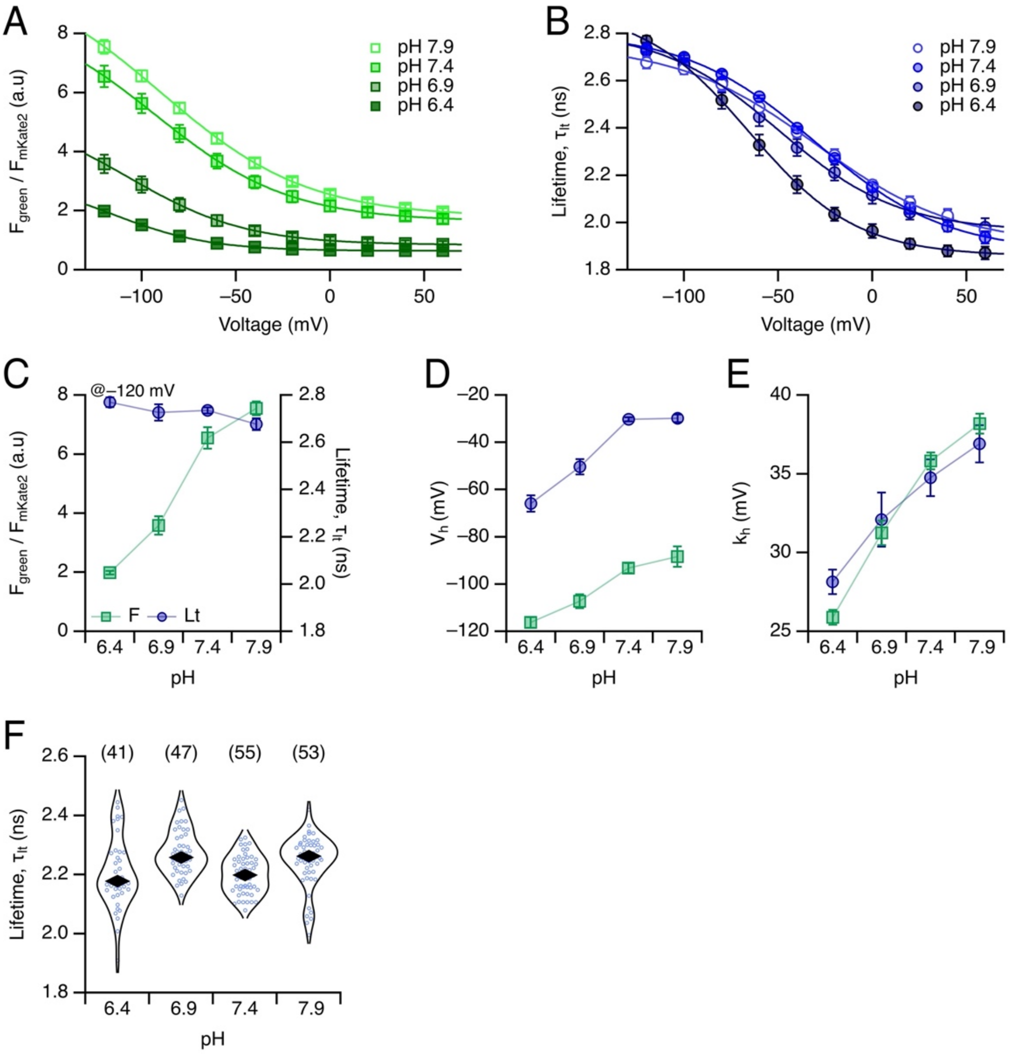

Supporting Figure 7. Dependence of rEstus-NI on extracellular pH. rEstus-NI was expressed in HEK293T cells. (A) Molecular brightness relative to the fluorescence of mKate2 (as in Figure S5) as a function of voltage for the indicated extracellular pH values. The intracellular (pipette) solution had pH 7.4 in all cases. Data are means ± SEM for *n* = 5 cells. The superimposed curves are data fits according to Eq. 1; the resulting parameters are listed in Table S3. (B) Fluorescence lifetime as a function of voltage of the same cells as in (A). (C-E) Parameters derived from the data shown in (A) and (B) as a function of pH: Relative brightness and fluorescence lifetime at -120 mV (C), half-maximal voltages, V_h (D), and the slope factors, k_h (E), for the fluorescence intensity (squares) and the fluorescence lifetime (circles). (F) Distributions of fluorescence lifetime values for fixed HEK293T cells expressing rEstus-NI in extracellular buffers with the indicated pH values. Each blue point corresponds to the median lifetime of an individual cell, and the black rhombi indicate the overall median of the cell medians for each condition. Cell fixation not only depolarizes the cells but also permeabilizes the membrane such that the indicated pH values also apply to the intracellular side.

Supporting Table 4. Voltage dependence of rEstus-NI fluorescence and fluorescence lifetime in HEK293T cells at different extracellular pH. The table contains the parameters describing the data fits shown in Figure S7. Errors are 95% confidence intervals.

pH	Parameter	Δa	a_{inf}	V_h (mV)	k_h (mV)	n
7.9	F_{green} / F_{mKate2}	8.38 ± 0.09	1.7 ± 0.01	-89.3 ± 0.8	38.7 ± 0.4	5
	Lifetime, τ_{lt}	0.85 ± 0.04 ns	1.91 ± 0.02 ns	-29.7 ± 2.1	37.1 ± 2.9	5
7.4	F_{green} / F_{mKate2}	7.23 ± 0.17	1.64 ± 0.01	-92.9 ± 1.7	35.8 ± 0.7	5
	Lifetime, τ_{lt}	0.93 ± 0.02 ns	1.88 ± 0.01 ns	-30.2 ± 0.9	34.8 ± 1.2	5
6.9	F_{green} / F_{mKate2}	4.54 ± 0.12	0.84 ± 0.01	-106.6 ± 1.7	31.3 ± 0.6	5
	Lifetime, τ_{lt}	0.85 ± 0.04 ns	1.96 ± 0.01 ns	-49.7 ± 2.3	31.9 ± 2.4	5
6.4	F_{green} / F_{mKate2}	2.51 ± 0.03	0.64 ± 0.01	-116.1 ± 0.6	26.0 ± 0.2	5
	Lifetime, τ_{lt}	1.05 ± 0.09 ns	1.86 ± 0.02 ns	-65.4 ± 5.6	28.7 ± 4.3	5

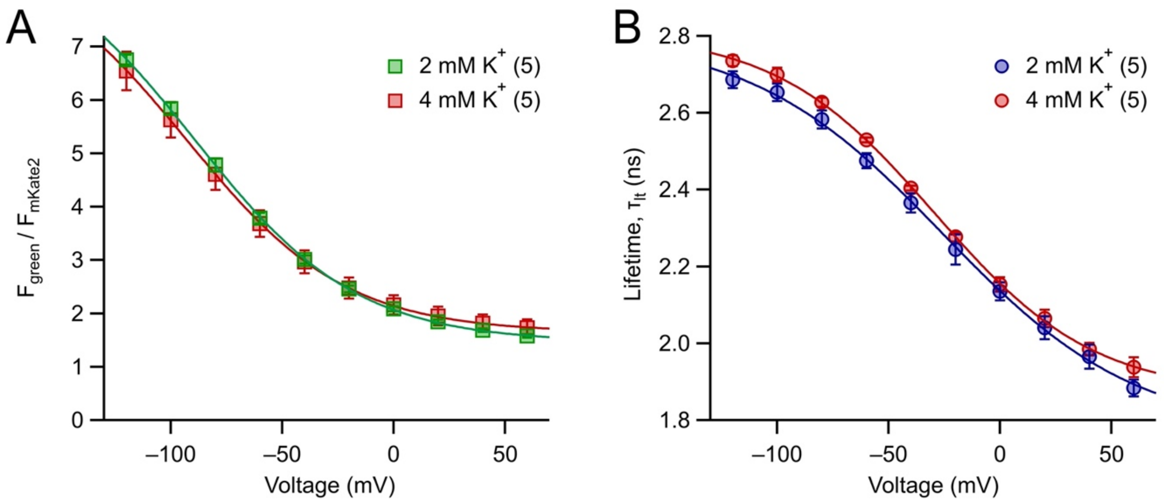

Supporting Figure 8. Calibration of rEstus-NI in 2-mM K^+ buffer. rEstus-NI was expressed in HEK293T cells and fluorescence intensity (A) and fluorescence lifetime (B) were measured under voltage-clamp control in the whole-cell configuration. Extracellular solutions either contained 4 mM or 2 mM K^+, as indicated. The superimposed curves are results of data fits according to Eq. 1, yielding the calibration parameters presented in Table S4.

Supporting Table 4. Voltage dependence of rEstus-NI fluorescence and fluorescence lifetime in HEK293T cells at different extracellular K^+ concentrations. The table contains the parameters describing the data fits shown in Figure S8. Errors are 95% confidence intervals.

$[K^+]_o$ (mM)	Parameter	Δa	a_{inf}	V_h (mV)	k_h (mV)	n
2	F_{green} / F_{mKate2}	7.60 ± 0.20	1.46 ± 0.02	-89.3 ± 1.8	36.5 ± 0.9	5
	Lifetime, τ_{lt}	1.04 ± 0.06 ns	1.77 ± 0.03 ns	-25.7 ± 2.6	43.2 ± 3.8	5
4	F_{green} / F_{mKate2}	7.23 ± 0.17	1.64 ± 0.01	-92.9 ± 1.7	35.8 ± 0.7	5
	Lifetime, τ_{lt}	0.94 ± 0.02 ns	1.87 ± 0.01 ns	-29.4 ± 1.7	35.8 ± 0.7	5

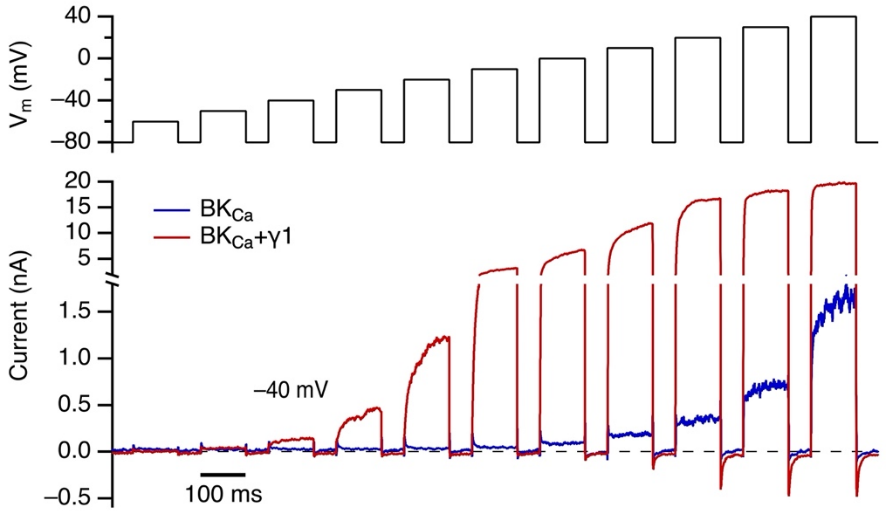

Supporting Figure 9. Function of BK_{Ca} and BK_{Ca}/BKγ1 channels in HEK293T cells. Whole-cell current recordings according to the pulse protocol shown in the top panel. HEK293T cell expressed rEstus-NI along with BK_{Ca} (Slo1, *KCNMA1*, blue) or BK_{Ca} together with BKγ1 (*KCNMA1-LRRC26*, red). Traces are means of three recordings. In the presence of BKγ1 current saturated at around 0 mV. The intracellular solution contained 100 nM free Ca^{2+}. Under these conditions, channel activity of BK_{Ca} alone is very low at -40 mV whereas in the presence of BKγ1 outward K^+ current is clearly discernable. Pipette solution in mM: 135 KCl, 2 $MgCl_2$, 3.93 $CaCl_2$, 10 EGTA, 10 HEPES, pH 7.4 (KOH).

Supporting Table 5. Voltage dependence of rEstus-NI fluorescence and fluorescence lifetime in human cancer cell lines. The table contains the parameters describing the data fits shown in Figure 4B for A375 and MCF-7 cells. Errors are 95% confidence intervals. Data for HEK293T cells are given as reference. In addition to the lifetime calibration based on the averages of lifetime frames, we also provide a calibration based on the medians to minimize the influence of extreme outliers.

Cell Type	Parameter	a_{max}	a_{inf}	V_h (mV)	k_h (mV)	n
A375	*F*	2.44 ± 0.07	1.92 ± 0.01	-104.6 ± 3.0	42.4 ± 1.2	5
	Lifetime, τ_{lt}	2.79 ± 0.02 ns	0.89 ± 0.03 ns	-36.8 ± 1.8	38.6 ± 2.4	5
median	Lifetime, τ_{lt}	2.79 ± 0.05 ns	0.94 ± 0.08 ns	-32.0 ± 4.5	39.0 ± 5.9	5
MCF-7	*F*	1.98 ± 0.07	1.53 ± 0.08	-79.4 ± 3.0	41.4 ± 1.2	5
	Lifetime, τ_{lt}	2.76 ± 0.01 ns	0.85 ± 0.03 ns	-24.0 ± 1.6	40.1 ± 2.2	5
median	Lifetime, τ_{lt}	2.74 ± 0.13 ns	0.84 ± 0.24 ns	-29.9 ± 13.1	40.8 ± 19	5
HEK293T	*F*	2.52 ± 0.09	2.09 ± 0.09	-93.4 ± 2.9	34.8 ± 1.4	5
	Lifetime, τ_{lt}	2.82 ± 0.02 ns	0.96 ± 0.03 ns	-29.2 ± 1.4	37.3 ± 1.9	5
median	Lifetime, τ_{lt}	2.81 ± 0.03 ns	0.93 ± 0.06 ns	-30.8 ± 2.7	34.7 ± 3.5	5

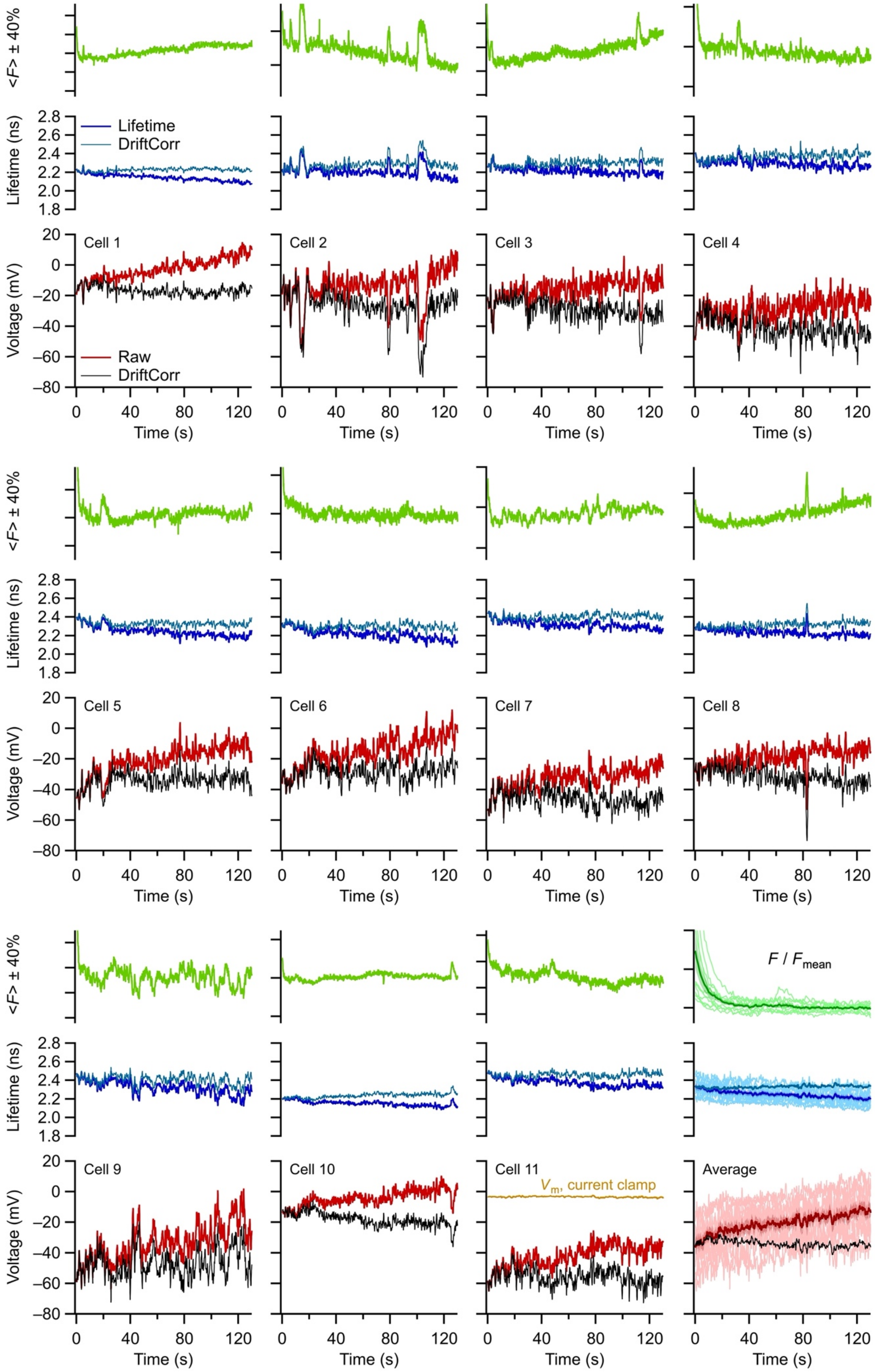

<F> ± 40%
Lifetime (ns)
Lifetime
DriftCorr
Voltage (mV)
Raw
DriftCorr
Cell 1
Cell 2
Cell 3
Cell 4
Cell 5
Cell 6
Cell 7
Cell 8
Cell 9
Cell 10
Cell 11
Average
V_m, current clamp
F / F_{mean}
Time (s)

Supporting Figure 10. Spontaneous V_m fluctuations in A375 cells. Time courses of FLIM recordings from individual rEstus-NI-transfected A375 cells with the fluorescence intensity (*top*) as mean fluorescence ± 40%, the fluorescence lifetime (*center*), and the voltage derived from the lifetime (*bottom*). The last panels (*bottom right*) display all traces (thin lines) with superimposed mean (thick line) and SEM in shading. Raw lifetime traces (dark blue) are also presented after drift correction (light blue) based on the mean drift in τ_{lt} measured under whole-cell voltage clamp at -40 mV. Similarly, the resulting voltage is shown based on raw lifetime traces (dark red) and on drift-corrected lifetimes (black). In the panels of the averaged traces, only the mean drift-corrected lifetime and voltage are presented in addition to the individual raw data. The orange trace in the voltage panel of Cell 11 is a voltage recording in current-clamp mode to illustrate the noise level when V_m is measured electrically with intracellular Ca^{2+} chelated with 10 mM EGTA.
